# DLREFD: a database providing associations of long non-coding RNAs, environmental factors and phenotypes

**DOI:** 10.1093/database/bax084

**Published:** 2017-10-25

**Authors:** Ya-Zhou Sun, De-Hong Zhang, Zhong Ming, Jian-Qiang Li, Xing Chen

**Affiliations:** 1 College of Computer Science and Software Engineering, Shenzhen University, Shenzhen 518060, China and; 2 School of Information and Control Engineering, China University of Mining and Technology, Xuzhou 221116, China

## Abstract

The development of many common complex diseases depends on the interactions between genetic factors (GF) and environmental factors (EF). Non-coding RNAs have been identified as major players in regulation of gene expression responding to environmental cues. In recent years, lots of studies have reported that the dysfunctions of long non-coding RNA (lncRNAs), EFs and their inter-actions have strong effects on phenotypes. However, compared with protein coding genes and microRNAs, there is a paucity of bioinformatics resource platform for understanding the disease mechanism in the level of lncRNA-EF interactions. In this study, we constructed the Disease Related LncRNA-EF Interaction Database (DLREFD), which contains a comprehensive collection and curation of experimentally supported interactions among lncRNAs, EFs and phenotypes. It integrated 835 entries, 475 LncRNAs, 153 EFs and 124 phenotypes. The names of lncRNAs, phenotypes, EFs, conditions of EFs, samples, species, evidence and references were further annotated. We hope DLREFD will be a useful resource for researches on lncRNAs, EFs and diseases.

**Database URL:** http://chengroup.cumt.edu.cn/DLREFD

## Introduction

Environmental factors (EF) can be any abiotic or biotic factors that influences living organisms (1). Abiotic factors include physical factors (e.g. heat shock, radiation, noise), chemical factors (e.g. small molecular drugs, complex compounds) and social factors (e.g. diet, stress, life style). Biotic factors would include variety of organisms such as parasites and viruses. It is well known that phenotypes of an organism are determined by the complex interactions between genetic factors and EFs. Apart from the true monogenic genetic disorders, EFs may determine the development of disease in those genetically predisposed to a particular condition. For example, stress, diet, pathogens, radiation and chemicals in personal-care products are common EFs that determine a large segment of non-hereditary disease. The majority of human complex diseases, such as cardiovascular disease, diabetes, and cancer, are caused by a combination of genetic and EFs (2).

Non-coding RNAs (ncRNAs) are a family of RNAs that display a variety of biochemically roles. During the past years, a large number of publication have documented plenty of important biological mechanisms and interaction patterns between microRNA (miRNA) and EFs. MiRNA have complex interactions with a wide spectrum of EFs including stress (3), drugs (4), virus (5), alcohol (6), air pollution (7), radiation (8), diet (9) etc. These interactions have crucial roles in many phenotypes including disease. Long non coding RNAs (lncRNAs) are defined as transcribed RNA molecules >200 nucleotides in length with no protein coding capability (10–12). In contrast to miRNAs, lncRNAs can fold into complex secondary and higher order structures, increasing the potential for both protein and target recognition. The role of lncRNAs in epigenetic processes has been recently highlighted. They have been demonstrated to control gene regulation at transcriptional level via DNA methylation and chromatin remodeling (13). They play important role as key regulators of health and disease and novel biomarkers of environmental exposure (14, 15). The developments in genomics and bioinformatics facilitated lncRNAs identification. LncRNA interacts with a variety of EFs such as environmental chemicals, cigarette smoking and air pollution (16, 17). They have been found to be related to a variety of human diseases that are known to include EFs as the causes in the etiology. For instance, the lncRNA HSR1 undergoes a structural conformational change in response to heat shock, stimulating the trimerization of the heat shock transcription factor 1 (HSF1), and thereby activating the process of heat shock response (18). Serum starvation results in an increase in GAS5 that functions as a repressor for glucocorticoid receptor (GR). Thus, it sensitizes human cells to cell death by environmental stressors (19). PRINS is increased by UV-B irradiation, viral infection and may contribute to psoriasis susceptibility (20). In addition, HOTAIR expression is induced after exposure to nanomolar concentrations of bisphenol A (BPA) in breast cancer cells (MCF7) (21). However, while lncRNAs have been found to be dysregulated in a variety of human disease that are known to include EFs in the etiology, compares to miRNA, little is still currently known about lncRNA interactions with environmental exposures, especially the further associations and regulating mechanisms between lncRNA and EFs. The studies on EFs and lncRNA associations are becoming increasing important in biomedical sciences. Therefore, a database linking lncRNAs, EFs and phenotypes becomes emergently needed but is still not available.

During the past years, several databases have been developed to provide comprehensive resources for associations between protein-coding gene/miRNA and EF, such as CTD and miREnvironment (22, 23). These databases greatly facilitate further research on the relationship between GF and EF. However, compared with protein-coding gene or miRNA, there is a paucity of databases linking lncRNA and EF. LncEnvironmentDB is a database to predict the associations between lncRNA and EF. However, it doesn’t contain the comprehensive collection of experimentally supported data of associations between lncRNA and EF (24). Therefore, a high-quality resource platform is believed to be of great value in the understanding of lncRNAs, EFs, especially drugs and diseases. More importantly, it will help to identified new biomarkers of diseases. Based on the high-quality data, the prediction of new associations between drugs and disease-related lncRNAs will promote drug repurposing and drug discovery.

In this paper, we describe the Disease Related LncRNA-Environmental Factor Interaction Database (DLREFD), a comprehensive online database established to collect the experimentally supported interactions among lncRNAs, EFs and phenotypes. We believe that this is the first database for disease-related lncRNA-EF associations. The database will help scientists and physicians in having an overview about the relationship between lnRNAs and EFs, and will be beneficial to understand the mechanism of lnRNA regulators in disease affected by EFs. Furthermore, DLREFD provides useful information for the diagnosis and prevention of diseases related to both genetic and EFs. The DLREFD can be publicly accessed from http://chengroup.cumt.edu.cn/DLREFD.

## Materials and methods

### Literature search and data extraction

DLREFD was designed to provide a web interface for users to browse and search datasets linking lncRNAs, EFs and phenotypes. To collect the experimentally supported associations, we searched the PubMed database for literature published before June, 23rd 2017 that matched this study by the union of two key-word sets. One keyword set is ‘long non-coding RNA or lncRNA’, which ensures that literature about lncRNA study is retrieved. The other keyword set contains a list of experimental factors according to the previous study (22) (Supplementary File S1). We further manually curated disease-related lncRNA-EF associations. Then we read the original references and manually retrieved the entries. The treated condition of EFs, samples, species, evidences describing the relationships and the reference PubMed ID were also manually collected. The data are further manually standardized and annotated. Items (i.e. lncRNA genes) that cannot be annotated are represented by ‘N/A’.

### Software design and implementation

Based on above datasets, we constructed the DLREFD database. In the DLREFD database, all datasets were organized in our web server using the browser/server framework based on PHP, Apache2 and MySQL system (25). The database is available at http://chengroup.cumt.edu.cn/DLREFD. DLREFD contains pages for browsing, searching, downloading and submitting.

## Results and discussion

### Data include in the database

**Figure 1.**
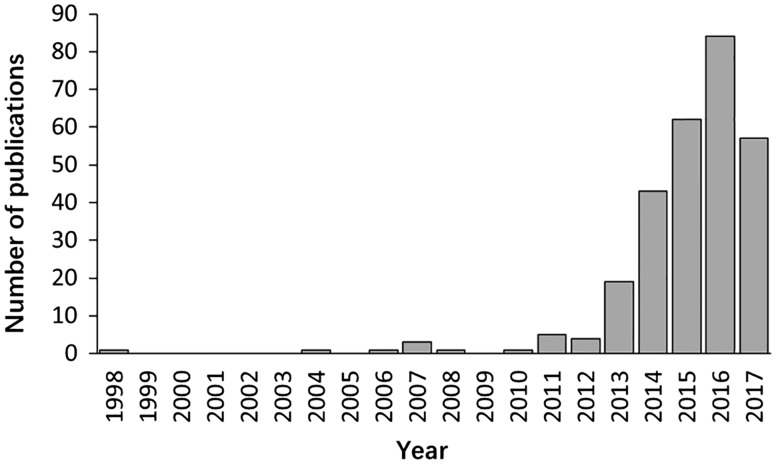
The distribution of papers included in the database by year of publication.

**Figure 2.**
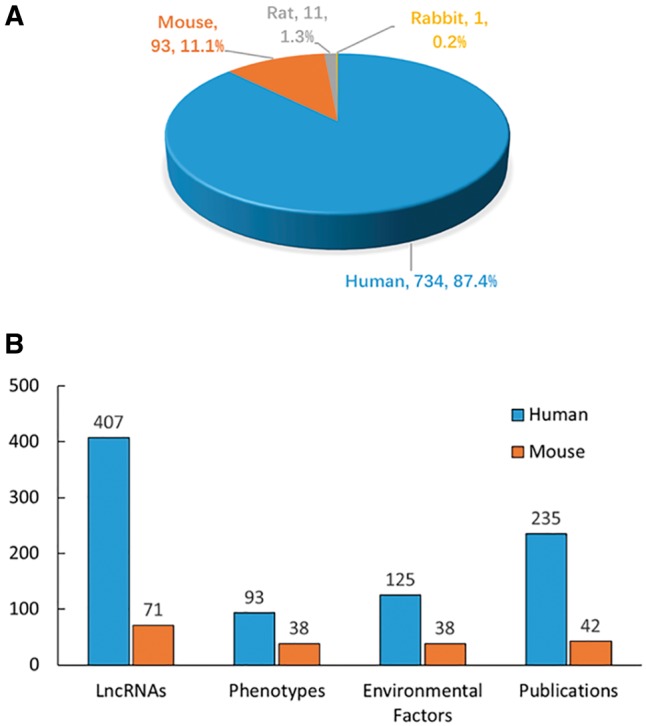
Statistics and distribution of data in DLREFD. **(A)** Entry distribution in different species. **(B)** Number of lncRNAs, phenotypes, EFs and publications for human and mouse.

The literature search yielded >6000 publications. To meet the need of DLREFD construction, we selected the literatures that provide complete information about lncRNA, EF and phenotype together. Importantly, the associations between lncRNAs and EFs must be verified by biological or clinical experiments. For example, if one study has identified certain EF associated lncRNAs by RNA-Seq, it should also provide evidence for the relationship by further experiments such as qRT-PCR, cell or animal model experiment. After filtering, the studies that not meet the inclusion criteria were rejected based on the title, abstract or the full text screening. Two hundred and eighty studies met the inclusion criteria and were included in the database. In the current version, DLREFD integrated 839 entries, 475 lncRNAs, 153 EFs, 124 phenotypes, 4 species from 280 publications. These relevant articles dated from 1998 to 2017 and the distribution of number of articles per year showed an increasing trend from 2012 (Figure 1). Human and mouse are the top two species that have the greatest numbers of entries. They represent 87.4% and 11.1% of the total entries (Figure 2A). We also list the statistical details for data of human and mouse (Figure 2B).

**Figure 3.**
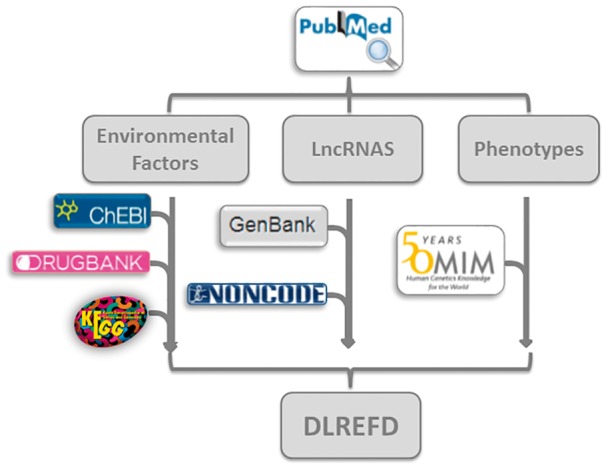
The flowchart of DLREFD construction. The ﬂowchart shows the process of data processing and information integration.

Every entry contains eight major items, which are lncRNA name, phenotype, EF, condition of EF, samples, species and the publication PubMed ID. The database also provides hyperlinks to the original references in NCBI (http://www.ncbi.nlm.nih.gov/) for each entry. We further annotated the drugs and compounds in EF from KEGG (26), DrugBank (27) and ChEBI (28). In all the 153 EFs, there are 111 typical drugs or chemical compounds. We have annotated 99 factors with at least one hyperlink. By linking to these databases, the users can easily know the features of interested drugs or compounds, such as chemical formula and structures, etc. In all 475 lncRNA sequences, 320 are annotated with information from Genbank (29) or NONCODE (30). And in 124 phenotypes, 66 are annotated by OMIM database (31) (Figure 3).

### The DLREFD web interface organization and functionality

**Figure 4.**
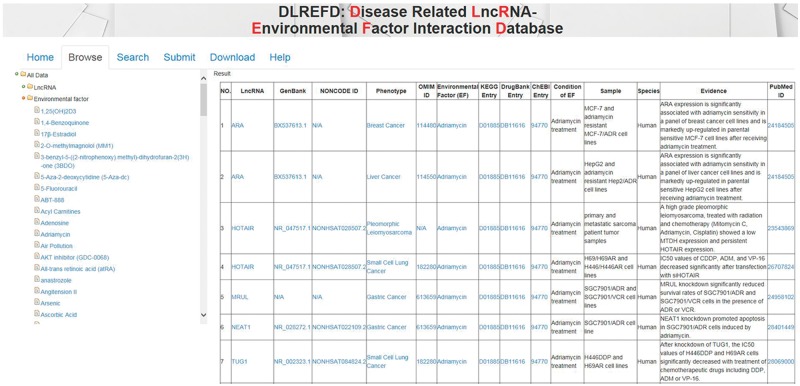
The NRDTD user interface showing the browse page.

The data in DLREFD can be easily accessed in various ways. First, users can browse the DLREFD by lncRNA, EF or phenotype names. To browse data in the database, select the menu ‘Browse’. And then select corresponding items to browse the entries you are interested. For example, if you want to get entries about the drug ‘Adriamycin’, you can click ‘environmental factors’ first and then select ‘Adriamycin’. The corresponding entries will be shown on the right panel (Figure 4). Second, we provided ‘search’ functions for the entries in the ‘Search’ page. To search data in the database, select the menu ‘Search’. DLREFD provides functions of ‘search’ by multiple keywords, such as LncRNA name, phenotype and EF name. Input your candidate keywords into corresponding blanks and submit the query. Moreover, all data in the database, including disease-related lncRNA–EF associations, descriptions of associations, publication PubMed ID, all lncRNA names, EF names and phenotype names, can be downloaded.

Aside from data retrieval from DLREFD, users can also submit novel data into the database. They may first search NRDTD to check whether their data have already been deposited into the database. If not, they can upload the related information. The novel entries will be forwarded to the DLREFD developers via email and will become available after a manual check and confirmation. A detailed tutorial for the usage of the database is available in the ‘Help’ page. In the future, the DLREFD will be updated continually and computational methods would be developed to predict novel disease related lncRNAs and EFs associations.

Using data in DLREFD, we can identify new disease indications for FDA-approved drugs, which is named drug repositioning. The drug-lncRNA interaction represents a novel dimension of information to drugs, and is expected to be useful in drug repositioning. For example, if we want to apply drug repositioning for gastric cancer, we can look at the related EFs including clinical drugs in DLREFD. Then we can try to combine them to develop synergistic drug combination. More importantly, we can search the ncRNAs related to gastric cancer and look at the drugs related to them. For instance, via the gastric cancer related lncRNA H19, we find related drug Temozolomide. Although Temozolomide is usually used in glioma and is not directly related to gastric cancer, it provides a new sight for us to test its efficiency to anti gastric cancer as they both related to lncRNA H19. In the future, we will also develop specific tools for prediction using network methods (32).

### Characteristics of lncRNA, EF and phenotype data

**Figure 5.**
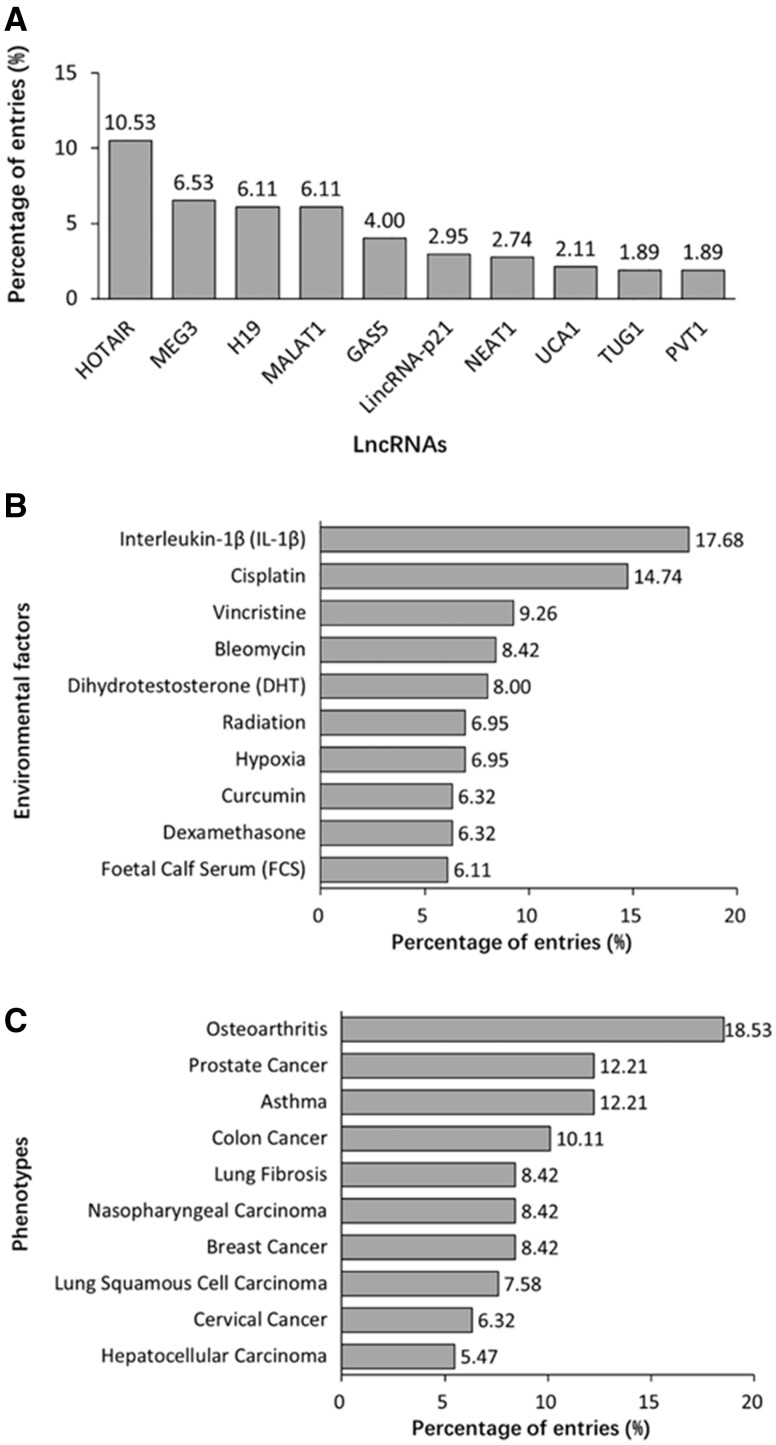
Summary statistics about lncRNA, EF and phenotype data registered in the database. **(A)** Distribution of top 10 lncRNAs. **(B)** Distribution of top 10 EFs. **(C)** Distribution of top 10 phenotypes.

A total of 475 lncRNAs are registered in DLREFD. Top 10 lncRNAs related to the most entries are shown in Figure 5A. The common feature of these lncRNAs is that they were identified earlier and their functions and mechanisms are studied deeply. However, benefiting from the developed sequencing technology, more and more new lncRNAs are identified and studied, which will expand the dataset of disease-related EF-affected lncRNAs. The top 10 EFs are shown in Figure 5B. Most of them are important compounds used in chemotherapy or method in radiotherapy, which indicted their significant roles in disease progress and treatment. Data analysis also reveals the top 10 phenotypes related to EF-lncRNA associations, such as osteoarthritis and asthma obviously have close relationships to EFs. Meanwhile, multiple cancers indicated that cancer is actually affected by both genetic and EFs (Figure 5C).

## Conclusion and future direction

Increasing studies have shown that lncRNAs have important functions and are involved in EF related human disease. In this article, we developed the DLREFD database focusing on disease related lncRNAs and EFs associations. At present, the number of entries in DLREFD is not very large. This is partly due to the experimental method of lncRNA study are time-consuming and most relationships among lncRNAs, EFs and phenotypes are uncertain. However, the important roles of lncRNAs in biomedical are attracting more scientific interest. As our understanding of mechanisms of ncRNAs improve, more disease-related lncRNAs-EF associations are expected to be reported and integrated into DLREFD. The purpose of DLREFD is to provide comprehensive resource about associations among lncRNA, EF and phenotype. Along with the number of associations in DLREFD increase consistently, DLREFD will become a more high-quality database for prediction of associations among lncRNA, EF and phenotype with perfect functions finally and make bigger contribution to solve actual biological problems.

We plan to update DLREFD every 2 months with the experimentally supported disease-related lncRNA-EF association data from newly published references. Meanwhile, some new tools for analysing association data is being developed and will be integrated into the DLREFD database in the future. For example, we will develop interacting similarity-based methods to predict novel disease-related lncRNA-EF association and expect to integrated these methods into database in the near future. We also plan to develop new tools based on gene expression data to analyse and quantify the effect of EF on lncRNAs. We believe that DLREFD would be useful for the studies of associations of lncRNAs, EFs and phenotypes, and will provide more helps when it integrates more data and tools in the future.

## Availability

DLREFD database is freely available at http://chengroup.cumt.edu.cn/DLREFD.

## Supplementary data

Supplementary data are available at *Database* Online.

## Supplementary Material

Supplementary File 1
